# TGF-β prevents the denervation-induced reduction of bone formation and promotes the bone regeneration through inhibiting ubiquitin-proteasome pathway

**DOI:** 10.1042/BSR20190350

**Published:** 2019-05-14

**Authors:** Zhen Yu, Ye Li, Yining Wang, Yuting Chen, Mengfan Wu, Zijue Wang, Minkai Song, Feng Lu, Xiaohe Lu, Ziqing Dong

**Affiliations:** 1 Department of Ophthalmology, Zhujiang Hospital, Southern Medical University, Guangzhou 515282, P. R. China; 2 Department of Plastic and Cosmetic Surgery, Nanfang Hospital, Southern Medical University, 1838 Guangzhou North Road, Guangzhou, Guangdong 510515, P. R. China; 3 Department of Orthopaedic Surgery, Nanfang Hospital, Southern Medical University, 1838 Guangzhou North Road, Guangzhou, Guangdong 510515, P. R. China

**Keywords:** Bone Formation, Dernervation, regeneration, RUNX2, TGF-β, ubiquitins

## Abstract

**Background:** Transforming growth factor beta (TGF-β) can stimulate osteogenesis as a multifunctional protein. The present study was to explore if TGF-β can prevent denervation-induced reduction of bone formation.

**Materials & methods:** The 6-week-old male mice were treated with recombinant human TGF-β1 (rhTGF-β1). Bone formation, endochondral bone growth rates, and gene expression of osteoblast markers were measured in the skeletal tissue by real-time PCR.

**Results:** RhTGF-β1 treatment prevented the denervation-induced decrease in bone formation rates, endochondral growth, and expression of Cbfa1/Runx2 (runt-related transcription factor 2), Ostecalcin (OC), and ColIA1. TGF-β1 partially inhibited the denervation-induced ubiquitination of Cbfa1/Runx2 in mouse cancellous bones via ubiquitin-proteasome pathway.

**Conclusion:** TGF-β prevents denervation-induced reduction of bone formation and promotes the bone regeneration through inhibiting ubiquitin-proteasome pathway at least partially.

## Introduction

Denervation results in bone loss in human and animal models [1,2]. Clinical and experimental studies indicate that bone metabolism becomes abnormal following denervation including markedly diminished bone density [3,4] and linear growth velocity [5]. In previous attempts to improve growth velocity in denervated-mice, recombinant human growth hormone, insulin-like growth factor binding protein (IGFBP), and insulin-like growth factor (IGF) were administered during the initial treatment [6]. However, it rarely attracts attention that the effects of local and systemic regulators on bone metabolism after denervation, including the activin/TGF-β/bone morphogenetic protein cytokine family which is important in stimulating bone regeneration.

TGF-β belongs the family of molecules with multifarious actions relative to bone metabolism [7]. There are several evidences indicated TGF-β1, 2, and 3 as having autocrine and/or paracrine roles in regeneration and remodeling of bone [8]. TGF-β stimulates chondrogenic differentiation [9] and increases cell proliferation [10], and chondrocytes [11] *in vitro*. Also, TGF-β promotes collagen forming [12] and collects osteoblast-like cells [13], and improves the wound of soft tissue after denervation [14] *in vivo*.

Administration of TGF-β promotes bone formation and osteogenesis at the injection site in experimental models. Local injections of TGF-β1 and TGF-β2 into the subperiosteal regions of either parietal or long bones resulted in stimulation of osteogenesis [15,16]. TGF-β is continuously expressed during fracture repair, and exogenous TGF-β affects dramatically on gene expression and differentiation of bone cells and cartilage [17]. Additionally, application of TGF-β *in vivo* can lead to the callus formation in normal bone, rapid closure of skull defects [18], and improve bone regeneration and strength during rat tibiofibular fractures repair [19]. Recent studies demonstrated thhypertrophyat TGF-β stimulates chondrocyte proliferation and differentiation [20]. In conclusion, TGF-β can regulate osteoclast and osteoblast function, but the role of endogenous TGF-β in bone formation and bone remodeling remains unclear. Therefore, the present study investigated the possibility that TGF-β may prevent bone loss after denervation.

## Materials and methods

### Animals and denervation

All experiments were approved by the Nanfang Hospital Animal Ethics Committee Laboratory (Guangzhou, P.R. China) and conducted according to the guidelines of the National Health and Medical Research Council of China. All experimental procedures were performed in Nanfang Hospital Experimental Animal Center (Guangzhou, P.R. China). Eighty-four 6-week-old male C57/BL6 mice weighing 16–18 g were obtained from the Southern Medical University (Guangzhou, P.R. China). The mice were maintained on a 12 h light/12 h dark cycle. All mice were randomly divided into four groups (twenty-one/group): Sham + Veh, Sham + TGF, DNV + Veh, and DNV + TGF. The mice were anesthetized by intraperitoneal injection of pentobarbital sodium (50 mg/kg). For DNV + Veh and DNV + TGF mice, the left nerve was ligated at approximately 1 cm proximal to the nerve trifurcation and removed out about 0.5 cm to prevent spontaneous regeneration. We performed sham surgeries to the Sham + Veh and Sham + TGF mice.

### Recombinant human TGF-β1 treatment and sampling

After the surgery, the Sham + Veh and DNV + Veh mice were treated by subcutaneous injection with vehicle and the Sham + TGF and DNV + TGF mice were administered using osmotic minipumps (Alza Corp., Palo Alto, CA, U.S.A.) with recombinant human TGF-β1 (rhTGF-β1) (R&D Systems, Minneapolis, MN, U.S.A.) 100 μm/kg daily at 2 h.

At week 1, 2, and 3, the mice were weighed and killed to sampling. Lumbar vertebral bodies and/or distal femurs were fixed in 70% ethanol and embedded in methyl methacrylate. A cross-section of the anterior portion of the vertebral body (15 μm) and the distal femur (4 and 15 μm) was prepared. The 4 μm sections were stained with toluidine blue, von Kossa’s silver nitrate, or modified Giemsa, while the 15 μm sections were not stained to evaluate of the fluorochrome bone markers.

### Morphometry

We used fluorochrome-based histomorphometric measurements of cancellous bone to determine the cross-sections of the lumbar vertebral bodies and frontal sections of the distal femurs in mice. All experiments have been repeated for three times. All of the morphometric parameters were calculated according to standard methods and were expressed in two-dimensional units. Measurements were performed on a digitized plate connected with a computer and an epifluorescence microscope using the morphometry program called “Stereology” (KSS Computer Engineers, Magna, UT, U.S.A.).

### Quantitative real-time PCR

The samples were quickly placed in a mortar precooled with liquid nitrogen. Repeatedly ground to a powder form in liquid nitrogen, and then transferred to a precooled homogenizer. The 1 ml of Trizol reagent (Invitrogen) was added, thoroughly homogenized, and centrifuged at 4°C (12000 r/min, 15 min). The supernatant was centrifuged with chloroform to separate RNA from cellular DNA, proteins and other components to obtain total RNA. RNA was suspended in RNase/DNase-free water (Gibco/Invitrogen, Carlsbad, CA) and quantified using an Agilent 2100 Bioanalyzer according to the RNA 6000 Nano Assay (Agilent Technologies, Palo Alto, CA). First-strand cDNA synthesis was performed using the SuperScript first-stand synthesis system for real-time PCR (Life Technologies, Rockville, MD) using oligo(dT) as a primer according to the manufacturer’s protocol. As an additional quality control, Arabidopsis thaliana mRNA was added to each RNA sample prior to cDNA synthesis. Real-time PCR was performed in a Smart Cycler (Cepheid, Sunnyvale, CA) using the LightCycler DNA Master SYBR Green I dye intercalation assay (Roche Molecular Biochemicals, Indianapolis, IN). Expression levels were calculated according to the 2^−ΔΔ*C*_t_^ method. Primers were generated as following: Cbfa1/Runx2 (runt-related transcription factor 2) (NM_009820), 5′-ATGCTTCATTCGCCTCACAAACAAC-3′ (sense) and 5′-ATTAACCATTTAAACGCCAGAG-3′ (antisense); Ostecalcin (OC) (X04142), 5′-AACAGACTCCGGCGCTACCTTG–3′ (sense) and 5′-AGCTCGTCACAAGCAGGGTTAAG-3′ (antisense); ColIA1(X54876), 5′-AGACGGGAGTTTCTCCTCGGGAC-3′ (sense) and 5′-TGTAGACTCTTTGCG GCTGGGGTG-3′ (antisense).

### Cell culture and Runx2 degradation assay

Mouse primary osteoblastic cell lines (MPOC) were cultured in RPMI-1640 medium (GIBCO, C11875500BT) supplemented with 10% fetal bovine serum (GIBCO, Cat. No. 10099-141) at 37°C and 5% CO_2_. For Runx2 degradation assay, MPOC were transiently transfected with pHA-Sunx2 expression vector by using Lipofectamine 2000 according to the instructions specified by the manufacturer (Invitrogen). The empty pcDNA3 vector was used as a negative control.

### Western blotting

Protein expression was detected by Western blotting according to the established protocols. The primary antibodies used were as follows: Anti-Runx2 (Oncogene, Cambridge, MA), anti-actin (sc-1616; Santa Cruz Biotechnology, Santa Cruz, CA), and anti-ubiquitin (sc-8017); anti-glceraldehyde-3-phosphate dehydrogenase (GAPDH, Ambion, Austin, TX). All images were captured and analyzed using a Tanon 5200 Chemiluminescent Imaging System (Tanon Science & Technology Co., Ltd, Shanghai, China), with densitometry analyses performed using ImageJ software (NIH, U.S.A.).

### Immunoprecipitation

Cancellous bone tissue lysates were centrifuged at 12000 × ***g*** for 10 min at 4°C. The resulting supernatants were collected for immunoprecipitation to assay the endogenous Runx2 degradation and *in vitro* Runx2 ubiquitination. The degradation assay was also performed in the presence of 1× proteasome inhibitor mix (0.25 mM MG132 and 0.25 mM MG115; EMD Biosciences, San Diego, CA, U.S.A.).

### Statistical analysis

Statistical analyses were performed using SPSS 24.0 (IBM SPSS, Armonk, NY, U.S.A.) software. Comparisons between groups were assessed using Wilcoxon test. The *P*<0.05 was considered statistically significant. Results are presented as mean ± standard deviations.

## Results

### rhTGF-β1 prevented denervation-induced reduction of bone formation in mice lumbar vertebral cancellous bone

**Figure 1:**
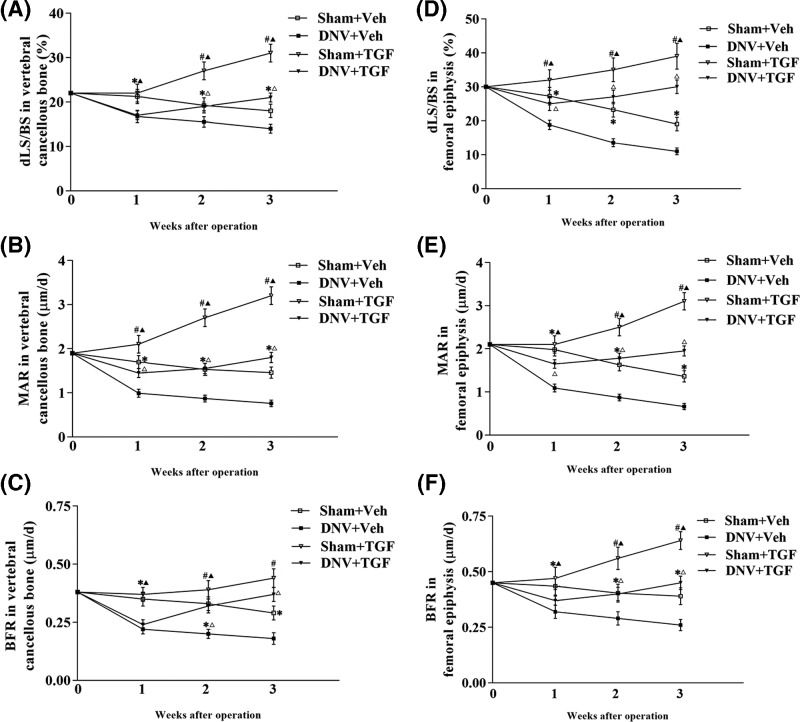
Effect of TGF-β1 on bone formation in lumbar vertebral and femoral epiphysis cancellous bone (**A**) Double-labeled surfaces/bone surface (dLS/BS), (**B**) Mineral apposition rate (MAR) and (**C**) Bone formation rate/bone surface (BFR/BS) in vertebral cancellous bone. (**D**) dLS/BS, (**E**) MAR, and (**F**) BFR/BS in femoral epiphysis. ^*^Sham + Veh vs DNV + Veh, *P*<0.05; ^#^Sham + Veh vs Sham + TGF, *P*<0.05. DNV + Veh vs DNV + TGF, *P*<0.05; Sham + TGF vs DNV + TGF, *P*<0.05. For all time points per group, *n*=7.

Double-labeled surfaces (dLS) (Figure 1A), mineral apposition rate (MAR) (Figure 1B), and bone formation rate (BFR) (Figure 1C) in the lumbar vertebral cancellous bone were significantly lower in DNV groups than in Sham groups at 1 week and were much lower at 3 weeks, reflecting denervation-induced severe reduction of bone formation as early as 1 week and throughout the experimental period. The rhTGF-β1 treatment prevented all those changes as early as at 1 week after denervation for MAR and BFR, and all reductions of dLS, MAR, and BFR were recovered to normal level at 2 weeks after denervation.

### RhTGF-β1 prevented denervation-induced reduction of bone formation in mice femoral epiphysis

A modest yet significant (30%, *P*<0.05) reduction in the single-labeled surface and a substantial decrease (42%, *P*<0.05) in the femoral epiphysis dLS in DNV groups were observed compared with the Sham groups (Figure 1D). Mineralizing surface and corrected MAR (Figure 1E) as well as surface and area referent BFR (Figure 1F) was significantly decreased (41%, *P*<0.05) in DNV groups at 1 week and were much lower at 3 weeks. Similar to the lumbar vertebral cancellous bone, rhTGF-β1 treatment prevented all those changes from 1 week DNV mice and reached to normal levels at 3 weeks in femoral epiphysis.

### RhTGF-β1 prevented denervation-induced reduction of osteoblast marker gene expression in mice femoral epiphyses

**Figure 2:**
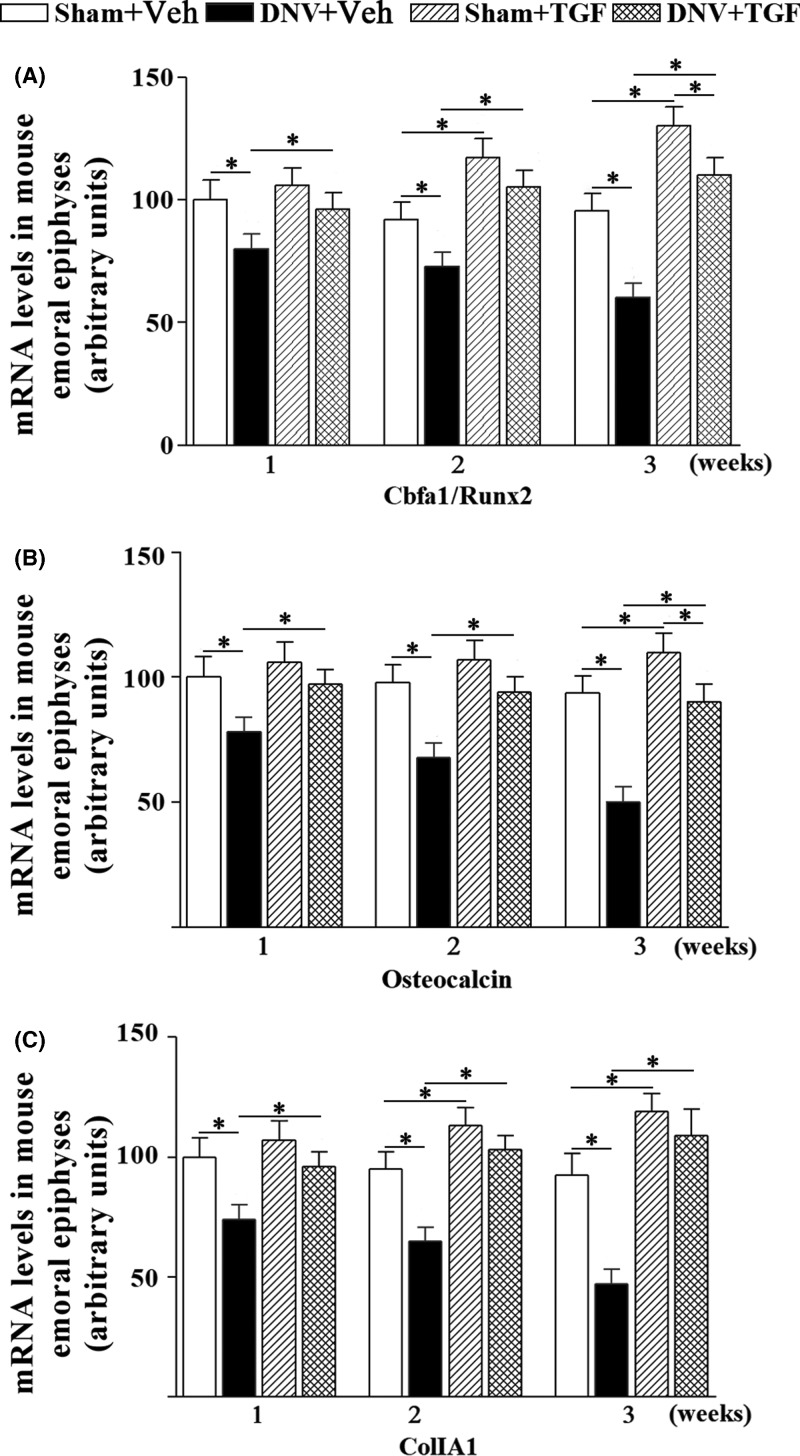
Effect of TGF-β1 on denervation-induced reduction of osteoblast marker gene expression in mice femoral epiphyses Quantitative analysis of mRNA expression for (**A**) Cbfa1/Runx2, (**B**) OC, and (**C**) ColIA1 gene in mice femoral epiphysis. ^*^P<0.05. For all time points per group, *n*=7.

**Figure 3:**
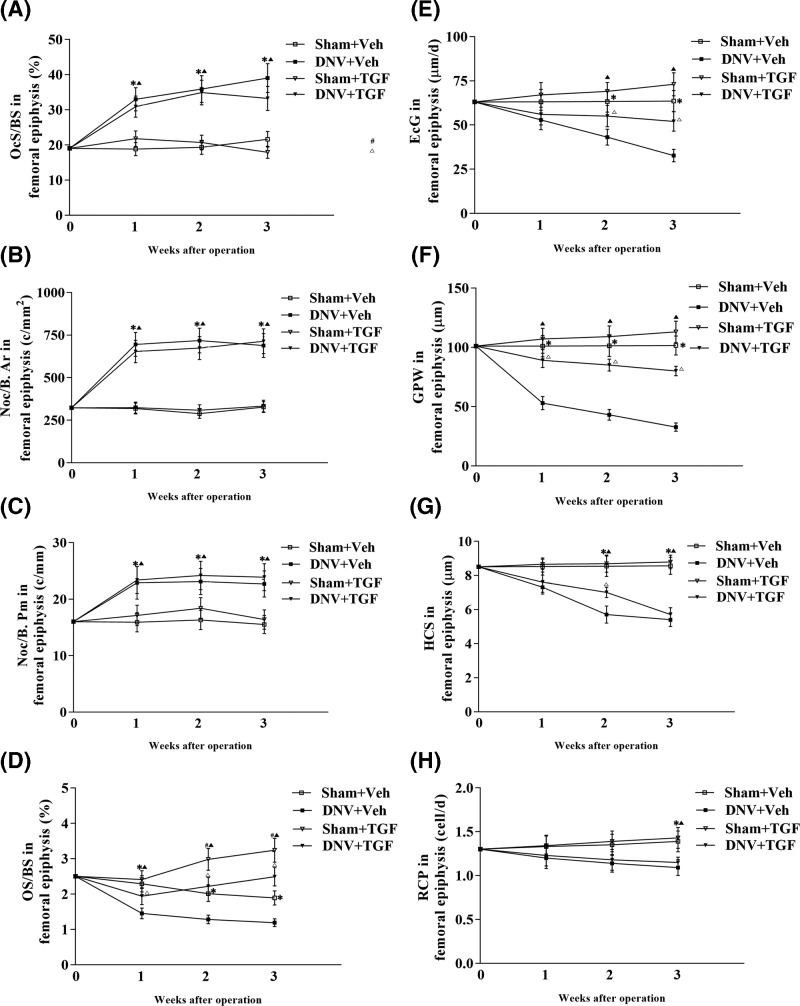
Effect of TGF-β1 on bone resorption in the proximal femoral metaphysis and endochondral growth indices of the proximal femurs (**A**) Osteoclast surfaces/bone surface (OcS/BS), (**B**) Number of osteoclast profiles/mm^2^ of cancellous bone (Noc/B. Ar), (**C**) Number of osteoclast profiles/mm of cancellous bone (Noc/B. Pm), (**D**) Osteoid surface/bone surface (OS/BS), (**E**) Endochondral growth (EcG), (**F**) Growth plate width (GPW), (**G**) Hypertrophic cell size (HCS) and (**H**) Rate of chondrocyte production (RCP) in femoral epiphysis. ^*^Sham + Veh vs DNV + Veh, *P*<0.05; ^#^Sham + Veh vs Sham + TGF, *P*<0.05. DNV + Veh vs DNV + TGF, *P*<0.05; Sham + TGF vs DNV + TGF, *P*<0.05. For all time points per group, *n*=7.

In femur, the expression of Cbfa1/Runx2 mRNA was significantly decreased, from 22 to 43% at week 1 and 3 in DNV groups compared with Sham groups (Figure 3A). With the treatment of TGF-β1, the Cbfa1/Runx2 mRNA level was elevated to as same as control group from 1 to 3 weeks (Figure 2A). Along with the change of Cbfa1/Runx2 mRNA in long-bone metaphyseal osteoblasts, the levels of OC mRNA decreased by 23, 30, and 50%, respectively (*P*<0.05) at week 1, 2, and 3 of DNV compared with Sham group (Figure 2B). TGF-β1 treatment for 1 week or late treatment for 2–3 weeks completely corrected the abnormal OC expression in metaphyseal bone of DNV + TGF group (Figure 2B). As same to the effects of denervation on Cbfa1/Runx2 mRNA and OC mRNA levels, the ColIA1 mRNA levels were reduced by 25, 29, and 53%, respectively (*P*<0.05) at week 1, 2, and 3 in metaphyseal bone compared with Sham groups. TGF-β1 treatment increased the ColIA1 mRNA levels to the normal as in Sham + TGF group at week 1–3 (Figure 2C). Meanwhile, TGF-β1 treatment increased the mRNA levels at week 2 and 3 for Cbfa1/Runx and ColIA1 and in week 3 for OC in Sham + TGF group. The results showed that exogenous TGF-β1 could restore the expression of osteoblast marker gene in the metaphyseal bone of DNV mice and promote normal bone formation.

### Effect of rhTGF-β on denervation-induced elevation of bone resorption in mice

A modest (34–37%, at week 1–3, *P*<0.05) decrease in the osteoid surface of DNV compared with Sham groups. There were significant differences in the numbers of nuclei per osteoclast or the numbers of osteoclasts per millimeter between Sham and DNV groups. However, the rhTGF-β1 treatment did not prevent denervation-induced increases in bone resorption although denervation-induced decrease in osteoid surface was prevented by rhTGF-β1 and even reached to the normal levels (Figure 3).

### RhTGF-β prevented denervation-induced reduction of endochondral growth in distal femurs of mice

**Figure 4:**
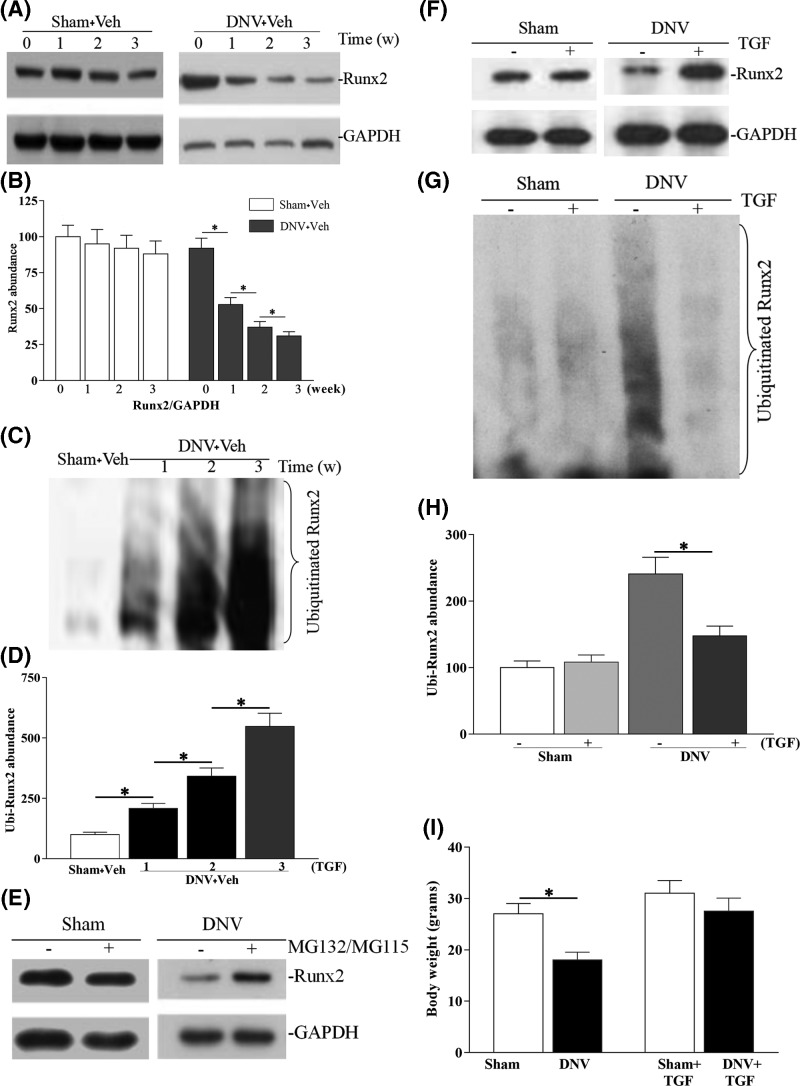
Effect of TGF-β1 on degradation and ubiquitination of Runx2 in denervated cancellous bone (**A**) Western blotting of Runx2 protein from denervated-cancellous bone of Sham + Veh and DNV + Veh groups. (**B**) Quantitative determination of Runx2 protein in (A). (**C**) The immunoprecipitated of HA-Runx2 protein in Sham + Veh and DNV + Veh groups. (**D**) Quantitative determination of the polyubiquitinated Runx2 abundance in (C). (**E**) Blockade of Runx2 protein degradation by the proteasome inhibitors. (**F**) Western blotting of Runx2 protein in denervated-cancellous bone from Sham + Veh, Sham + TGF, DNV + Veh, and DNV + TGF groups at 3 weeks. (**G**) The immunoprecipitated of exogenous Runx2 in the denervated-cancellous bone from Sham + Veh, Sham + TGF, DNV + Veh, and DNV + TGF groups at 3 weeks. (**H**) Quantitative determination of the poly-ubiquitinated Runx2 abundance shown in (G). (**I**) Total body weight in four groups. ^*^P<0.05. For all time points per group, *n*=7.

A significant decrease in endochondral growth (EcG) indices, including growth plate width (GPW), EcG, calculated rate of chondrocyte production (RCP) and hypertrophic cell size in DNV-mice compared with Sham-mice (Figure 3). These animals grew rapidly, with distal femoral endochondral bone elongation rates of 28 μm/d for DNV-mice and 53 μm/d for Sham-mice at 3 weeks. DNV-mice had final body weights of 18 ± 2 g compared with 27 ± 3 g for Sham group (Figure 4I). Interestingly, treatment with rhTGF-β1 resulted in increases significantly in longitudinal GPW and growth rate, and returned body weights in DNV group to the normal (Sham group) level. However, rhTGF-β1 treatment did not show any effects on RCP and hypertrophy cell size in both Sham and DNV groups. Examination of vertebrae examined under polarized light revealed that bone formation in the rhTGF-β1 treated group was layered rather than woven bone (Supplementary Materials).

### Denervation increased degradation of Runx2 in the cancellous bone

According to the *in vitro* degradation assay, degradation of Runx2 protein increased in the denervated cancellous bone, compared with the Sham group (Figure 4A). More than 70% of Runx2 protein vanished after a 16-h incubation (Figure 4B). In this case, only 15% of Runx2 protein disappeared in Sham groups. In addition, the results showed that proteasome inhibitors highly prevent denervation-induced Runx2 degradation in cancellous bone (Figure 4E). It suggested that denervation-induced the reduction of bone formation may through increasing proteasome-mediated degradation of Runx2 protein in the cancellous bone.

### Denervation promotes Runx2 ubiquitination in the cancellous bone

As shown in Figure 4C, for the cancellous bone, anti-ubiquitin antibodies detected multiple bands of Runx2 protein that were significantly poly-ubiquitinated in the reaction using homogenate (lanes 2–4). In contrast, only a fuzzy band was observed in the samples of the Sham groups (lane 1). Quantitative measurements showed that, at 3 weeks after denervation, the poly-ubiquitination Runx2 abundance of cancellous bone homogenate was five-times greater than that of the Sham group (Figure 4D), which suggested that ubiquitination activity for Runx2 protein is enhanced in the denervated cancellous bone.

### TGF-β1 prevents the Runx2 degradation and ubiquitination in denervated cancellous bone

We tested the Runx2 degradation and ubiquitination levels in sham and DNV cancellous bone with or without TGF-β1 treatment at 3 weeks. In the absence of TGF-β1 treatment DNV group, Runx2 abundance obviously reduced about 80% compared with sham (Figure 4F). However, TGF-β1 treatment markedly prevented Runx2 protein degradation in denervated cancellous bone. Similarly, TGF-β1 treatment largely prevented Runx2 ubiquitination in denervated cancellous bone (Figure 4G,H). It suggested that TGF-β1 prevents the denervation-induced the reduction of bone formation may be via inhibiting Runx2 protein degradation through ubiquitin-proteasome pathway.

## Discussion

The use of growth factors to stimulate bone formation provides a possible treatment for patients with denervation. In clinical and experimental, most of the therapies are based on the stimulation of bone formation, but inhibition of bone resorption still needs further research. TGF-β has a strong regulatory effect in both bone resorption and formation, and the sufficient evidences demonstrate that TGF-β plays an important role in osteogenesis and chondrogenesis [7,10,12,15,18]. In the present study, we describe that TGF-β prevents denervation-induced reduction of bone formation in mice over the short term.

The dramatic recovery of BFR observed following TGF-β treatment in DNV-mice can be attributed to a significant inhibition of the reduction in mineral appositional rate, which means that rhTGF-β1 treatment stimulates osteoblasts produce bone matrix. Several *in vitro* studies have shown that TGF-β can increase the collagen-forming ability of osteoblasts [10,12,15] and improve bone matrix attachment to bone organ culture [21].

In DNV groups, rhTGF-β1 treatment inhibited reduction the dLS, which suggested TGF-β prevented a decrease in the number of active osteoblasts. @@It is unknown that which TGF-β exerts its action. However, according to these rapid responses, the administration of rhTGF-β1 stimulated mitogenic differentiation of osteoblast progenitor cells or accelerated differentiation of already committed osteoblast progenitor cells. Due to the chemotactic response of TGF-βstimulated to osteoblast-like cells [13], the mitogenic responses to TGF-β may also be involved. Mitogenesis, accelerated differentiation of osteogenic precursors and chemotaxis of osteoblasts are all theoretical sequelae of rhTGF-β1. More research is needed to ascertain the responsible phenomena for the rapid recovery of BFR in denervated-mice after the initiation of TGF-β observed in the present study.

TGF-β has been found in osteoclasts [22]. It has be reported that TGF-β promotes resorption in cultured neonatal mouse calvarias by a prostaglandin-dependent mechanism [23]. However, evidence exists both for and against a role for prostaglandins in bone formation following denervation [23,24]. Thus, the effect of TGF-β is complex on osteoclasts and best studied *in vivo*. The exact mechanism of TGF-β activity is still unknown.

In recent years, research on TGF-β and bone marrow stem cells (BMSCs) has been increasing. In fact, the TGF-β related pathways could regulate almost all aspects of BMSCs function [25]. For example, MiR-663 inhibited the proliferation and migration of BMSCs by targeting TGF-β1 [26], and MOTS-c promoted cell differentiation of BMSCs to osteoblasts via TGF-β/Smad pathway [27]. Also study showed that HFD disrupted the TGF-β receptor within lipid rafts, associated with impaired Smad2/3-dependent TGF-β signaling [28]. Thus BMSCs might play an important role in the process of TGF-β increasing bone regeneration, especially the cell differentiation of BMSCs to osteoblasts.

Several researches reported that the additional osteoclasts were collected to reshape the TGF-β induced bone, which was not directly induced by TGF-β [29,30]. With rhTGF-β administration of short periods, as the present study, we did not observe a significant recovery in bone volume, nuclei per cell profiles, and the number of osteoclast per millimeter. When decreased BFR are sustained with longer periods of TGF-β treatment, increased bone mass should be observed. While, with the significant recovered levels in BFR observed here, no corresponding recovery in bone mass was observed. This complicated issue needs further study.

Denervation-induced increases in bone resorption may contribute to increased production of endogenous corticosteroids [31], impairing osteoblast recruitment and function. Our results, however, did not demonstrate rhTGF-β prevention of the denervation-induced increase in bone resorption, and no report exists indicating an interaction between glucocorticoids and rhTGF-β.

Endochondral bone formation includes a cascade of cellular events. Prevention by rhTGF-β1 of denervation-induced reduction of endochondral bone formation may stimulate chondrocyte proliferation and chondrogenic differentiation via a complex network of endogenous growth/differentiation signaling molecules, including the Indian hedgehog/parathyroid hormone-related peptide (Ihh/PTHrP) feed-loop pathway. There is evidence showing that TGF-β up-regulates the Ihh and PTHrP gene expression in chondrocyte and both genes stimulate chondrocyte proliferation and chondrogenic differentiation [32]. The molecular mechanism by which TGF-β prevents denervation-induced reduction of endochondral bone formation requires further exploration.

Runx2 has been found to play an important role in the regeneration of various tissues recently years [33–36]. Especially the role of Runx2 in bone tissue regeneration is particularly popular. Such as the high-fat and high-glucose microenvironment inhibited bone regeneration, which was related to the inhibition of Runx2 expression [37]. Also in the bone fractures and postmenopausal osteoporosis inhibiting bone regeneration, Runx2 expression was reduced [36,38]. In our study, we found the bone regeneration was inhibited in the DNV group, and also the expression of Runx2 was decreased. When treated with TGF-β, the inhibiting of bone regeneration was decreased and the expression of Runx2 was increased. Thus the Runx2 might be a potential drug target, which could be used to increase the bone regeneration in multiple diseases clinically.

Denervation induced a significant decrease in the expression of Cbfa1 / Runx2 (a transcriptional activator of osteoblast differentiation), consistent with the reduction in bone formation. This is related to a decrease in the expression of the ColIA1 and OC genes known to be partially controlled by the Cbfa1/Runx2 in postnatal organisms [39]. Thus, the loss of Cbfa1/Runx2 expression may contribute to a decrease in ColIA1 and OC expression in denervated osteoblasts together with other transcriptional activators. Here, exogenous addition of TGF-β1 into denervated-mice can reduce the formation of defective bone. TGF-β1 treatment was related to the increase of Cbfa1/Runx2 expression, and the increase of ColIA1 and OC mRNA levels in denervated-mice, suggesting that TGF-β1 acts by increasing these target genes expression in metaphyseal osteoblasts. While the correction of osteoblastic gene expression also may be affected by post-transcriptional effects of TGF-β1 [40]. Our current study showed TGF-β inhibited denervation-induced Runx2 protein degradation and ubiquitination, but whether denervation increases OC and ColIA1 protein degradation and/or ubiquitination; TGF-β affects it; if so whether it is through runx2 regulation and/or through ubiquitin-proteasome pathway, further studies are necessary to clarify this point.

Protein degradation is essential for controlling many cellular processes. The ubiquitin-proteasome pathway consists of a highly organized cascade of enzymatic reactions and requires energy, which can sequence, label and destroy a variety of physiologically important proteins [41]. The explanation of protein substrates caused by ubiquitin-proteasome proteolytic pathway defects is associated with many devastating disorders [41–43]. Under both physiologic and pathologic conditions, the Smad signaling pathway༌ including Runx2༌ undergo ubiquitin-proteasome mediated degradation. Increased proteasomal degradation and ubiquitination against Runx2 in denervated-cancellous bone means that Runx2 specifically targets ubiquitin-dependent destruction *in vivo* during bone-remodeling. Studies suggest that TGF-β1 can inhibit Runx2 degradation in denervated cancellous bone (Figure 4). This is consistent with previous studies that TGF-β1 also is shown to reduce matrix degradation [44]. These observations suggest that changes in key signaling regulators in ubiquitin-proteasome degradation may be due to interference with important cellular signaling, which in turn leads to osteoblast dysfunction and contributes to the pathogenesis of bone formation. Functionally, stabilization of Runx2 by the TGF-β1 leads to an inhibition denervation–induced reduction of bone formation gene expression. Therefore, these findings establish that TGF-β regulating protein degradation may be via ubiquitin-proteasome pathway and may play an imperative role in the pathogenesis of denervation–induced reduction of bone formation.

## Conclusion

In summary, we report that TGF-β prevents denervation-induced reduction of bone formation for short periods of time by inhibiting its decreasing mRNA level of Cbfa1/Runx2, OC and ColIA1, and ubiquitination of Runx2. The precise role of the TGF-β superfamily in injury-induced skeletal changes in denervated-mice is unknown. It is not certain whether rh TGF-β acts directly on osteoblasts/chondrocytes or their progenitor cells. Other endocrine, paracrine and/or autocrine media may also be involved. We report here that TGF-β1 prevents the denervation-induced reduction of bone formation, suggesting a mechanism involving TGF-β1 in the altered differentiation of osteoblasts in denervated-mouse bone.

## Supporting information

**Supplementary Materials:** 

## Abbreviations

BFR: bone formation rate
CollA1: Collagen type I alpha1
DLS: Double-labeled surfaces
DNV: Denervation
EcG: endochondral growth
GPW: growth plate width
MAR: mineral apposition rate
MPOC: mouse primary osteoblastic cell lines
OC: Ostecalcin
rhTGF-β1: recombinant human TGF-β1
Runx2: runt-related transcription factor 2
TGF-β: transforming growth factor beta

## References

[1] Lerner U. (2006) Deletions of genes encoding calcitonin/alpha-CGRP, amylin and calcitonin receptor have given new and unexpected insights into the function of calcitonin receptors and calcitonin receptor-like receptors in bone. J. Musculoskelet. Neuronal. Interact. 6, 87–95 16675892

[2] Le P.T., Bishop K.A., Maridas D.E., Motyl K.J., Brooks D.J., Nagano K. (2017) Spontaneous mutation of Dock7 results in lower trabecular bone mass and impaired periosteal expansion in aged female Misty mice. Bone 105, 103–114 10.1016/j.bone.2017.08.006 28821457 PMC5693233

[3] Pagani F., Sibilia V., Cavani F., Ferretti M., Bertoni L., Palumbo C. (2008) Sympathectomy alters bone architecture in adult growing rats. J. Cell. Biochem. 104, 2155–2164 10.1002/jcb.21775 18449939

[4] Gargiulo P., Reynisson P.J., Helgason B., Kern H., Mayr W., Ingvarsson P. (2011) Muscle, tendons, and bone: structural changes during denervation and FES treatment. Neurol. Res. 33, 750–758 10.1179/1743132811Y.0000000007 21756556

[5] Li Y., Jie L., Tian A.Y., Zhong S., Tian M.Y., Zhong Y. (2017) Transforming growth factor beta is regulated by a glucocorticoid-dependent mechanism in denervation mouse bone. Sci. Rep. 7, 9925 10.1038/s41598-017-09793-y 28855536 PMC5577242

[6] Suzue N., Nikawa T., Onishi Y., Yamada C., Hirasaka K., Ogawa T. (2006) Ubiquitin ligase Cbl-b downregulates bone formation through suppression of IGF-I signaling in osteoblasts during denervation. J. Bone Miner. Res. 21, 722–734 10.1359/jbmr.060207 16734387

[7] Patil A., Sable R. and Kothari R. (2011) An update on transforming growth factor-β (TGF-β): sources, types, functions and clinical applicability for cartilage/bone healing. J. Cell. Physiol. 226, 3094–3103 10.1002/jcp.22698 21344394

[8] Sykaras N. and Opperman L.A. (2003) Bone morphogenetic proteins (BMPs): how do they function and what can they offer the clinician? J. Oral. Sci. 45, 57–73 10.2334/josnusd.45.57 12930129

[9] Li Y., Tian A.Y., Ophene J., Tian M.Y., Yao Z., Chen S. (2017) TGF-β stimulates endochondral differentiation after denervation. Int. J. Med. Sci. 14, 382–389 10.7150/ijms.17364 28553171 PMC5436481

[10] Centrella M., McCarthy T. and Canalis E. (1987) Transforming growth factor beta is a bifunctional regulator of replication and collagen synthesis in osteoblast-enriched cell cultures from fetal rat bone. J. Biol. Chem. 262, 2869–2874 3469200

[11] Engstrand T. (2003) Molecular biologic aspects of cartilage and bone: potential clinical applications. Ups. J. Med. Sci. 108, 25–35 12903835

[12] Liu X., Long X., Liu W., Zhao Y., Hayashi T., Yamato M. (2018) Type I collagen induces mesenchymal cell differentiation into myofibroblasts through YAP-induced TGF-β1 activation. Biochimie 150, 110–130 10.1016/j.biochi.2018.05.005 29777737

[13] Dobolyi A., Vincze C., Pál G. and Lovas G. (2012) The neuroprotective functions of transforming growth factor beta proteins. Int. J. Mol. Sci. 13, 8219–8258 10.3390/ijms13078219 22942700 PMC3430231

[14] Xu X., Zheng L., Yuan Q., Zhen G., Crane J.L., Zhou X. (2018) Transforming growth factor-β in stem cells and tissue homeostasis. Bone Res. 6, 2 10.1038/s41413-017-0005-4 29423331 PMC5802812

[15] Noda M. and Camilliere J. (1989) *In vivo* stimulation of bone formation by transforming growth factor-beta. Endocrinology 124, 2991–2994 10.1210/endo-124-6-2991 2721454

[16] Zellin G. (1998) Growth factors and bone regeneration. Implications of barrier membranes. Swed. Dent. J. Suppl. 129, 7–65 9672999

[17] Cho T., Gerstenfeld L. and Einhorn T. (2002) Differential temporal expression of members of the transforming growth factor beta superfamily during murine fracture healing. J. Bone Miner. Res. 17, 513–520 10.1359/jbmr.2002.17.3.513 11874242

[18] Beck L., Deguzman L., Lee W.P., Xu Y., McFatridge L.A., Gillett N.A. (1991) Rapid publication. TGF-beta 1 induces bone closure of skull defects. J. Bone Miner. Res. 6, 1257–1265 10.1002/jbmr.5650061117 1805548

[19] Nielsen H., Andreassen T.T., Ledet T. and Oxlund H. (1994) Local injection of TGF-beta increases the strength of tibial fractures in the rat. Acta Orthop. Scand. 65, 37–41 8154281 10.3109/17453679408993715

[20] Lin Z., Navarro V.P., Kempeinen K.M., Franco L.M., Jin Q., Sugai J.V. (2010) LMP1 regulates periodontal ligament progenitor cell proliferation and differentiation. Bone 47, 55–64 10.1016/j.bone.2010.03.013 20348040 PMC2891403

[21] Gazit D., Zilberman Y., Turgeman G., Zhou S. and Kahn A. (1999) Recombinant TGF-beta1 stimulates bone marrow osteoprogenitor cell activity and bone matrix synthesis in osteopenic, old male mice. J. Cell. Biochem. 73, 379–389 10.1002/(SICI)1097-4644(19990601)73:3<379::AID-JCB9>3.0.CO;2-U 10321837

[22] Shakir S., MacIsaac Z.M., Naran S., Smith D.M., Bykowski M.R., Cray J.J. (2015) Transforming growth factor beta 1 augments calvarial defect healing and promotes suture regeneration. Tissue Eng. Part A 21, 939–947 10.1089/ten.tea.2014.0189 25380311 PMC4356478

[23] Ghayor C., Rey A. and Caverzasio J. (2005) Prostaglandin-dependent activation of ERK mediates cell proliferation induced by transforming growth factor beta in mouse osteoblastic cells. Bone 36, 93–100 10.1016/j.bone.2004.10.007 15664007

[24] Chenu C., Kurihara N., Mundy G.R. and Roodman G.D. (1990) Prostaglandin E2 inhibits formation of osteoclastlike cells in long-term human marrow cultures but is not a mediator of the inhibitory effects of transforming growth factor beta. J. Bone Miner. Res. 5, 677–681 10.1002/jbmr.5650050703 2396495

[25] Su J., Zhang L., Zhang W., Choi D.S., Wen J., Jiang B. (2014) Targeting the biophysical properties of the myeloma initiating cell niches: a pharmaceutical synergism analysis using multi-scale agent-based modeling. PLoS ONE 9, e85059 10.1371/journal.pone.0085059 24475036 PMC3903473

[26] Geng L., Tang X., Zhou K., Wang D., Wang S., Yao G. (2019) MicroRNA-663 induces immune dysregulation by inhibiting TGF-β1 production in bone marrow-derived mesenchymal stem cells in patients with systemic lupus erythematosus. Cell. Mol. Immunol. 16, 260–274 10.1038/cmi.2018.1 30886422 PMC6460486

[27] Hu B.T. and Chen W.Z. (2018) MOTS-c improves osteoporosis by promoting osteogenic differentiation of bone marrow mesenchymal stem cells via TGF-β/Smad pathway. Eur. Rev. Med. Pharmacol. Sci. 22, 7156–7163 30468456 10.26355/eurrev_201811_16247

[28] Hermetet F., Buffière A., Aznague A., Pais de Barros J.P., Bastie J.N. (2019) Delva LHigh-fat diet disturbs lipid raft/TGF-β signaling-mediated maintenance of hematopoietic stem cells in mouse bone marrow. Nat. Commun. 10, 523 10.1038/s41467-018-08228-0 30705272 PMC6355776

[29] Marcelli C., Yates A. and Mundy G. (1990) *In vivo* effects of human recombinant transforming growth factor beta on bone turnover in normal mice. J. Bone Miner. Res. 5, 1087–1096 10.1002/jbmr.5650051013 2080720

[30] Islamov R., Chintalgattu V., Pak E.S., Katwa L.C. and Murashov A.K. (2004) Induction of VEGF and its Flt-1 receptor after sciatic nerve crush injury. Neuroreport 15, 2117–2121 10.1097/00001756-200409150-00024 15486493

[31] Bigbee A., Grindeland R.E., Roy R.R., Zhong H., Gosselink K.L., Arnaud S. (2006) Basal and evoked levels of bioassayable growth hormone are altered by hindlimb unloading. J. Appl. Physiol. 100, 1037–1042 10.1152/japplphysiol.00615.2005 16339349

[32] Komori T. (2002) Cbfa1/Runx2, an essential transcription factor for the regulation of osteoblast differentiation. Nippon Rinsho 60, 91–97 11979975

[33] Franceschi R.T. and Xiao G. (2003) Regulation of the osteoblastspecific transcription factor, Runx2: responsiveness to multiple signal transduction pathways. J. Cell. Biochem. 88, 446–454 10.1002/jcb.10369 12532321

[34] Sevetson B., Taylor S. and Pan Y. (2004) Cbfa1/RUNX2 directs specific expression of the sclerosteosis gene(SOST). J. Biol. Chem. 279, 13849–13858 10.1074/jbc.M306249200 14739291

[35] Jun J.H., Yoon W.J., Seo S.B., Woo K.M., Kim G.S., Ryoo H.M. (2010) BMP2-activated Erk/MAP kinase stabilizes Runx2 by increasing p300 levels and histone acetyltransferase activity. J. Biol. Chem. 285, 36410–36419 10.1074/jbc.M110.142307 20851880 PMC2978570

[36] Li P., Kong J., Chen Z., Huang S., Lv G., Wei B. (2019) Aloin promotes osteogenesis of bone-marrow-derived mesenchymal stem cells via the ERK1/2-dependent Runx2 signaling pathway. J. Nat. Med. 73, 104–113 10.1007/s11418-018-1249-z 30218208

[37] Wu X., Zhang Y., Xing Y., Zhao B., Zhou C. (2019) Wen YHigh-fat and high-glucose microenvironment decreases Runx2 and TAZ expression and inhibits bone regeneration in the mouse. J. Orthop. Surg. Res. 14, 55 10.1186/s13018-019-1084-2 30777111 PMC6380030

[38] Shan Y., Wang L., Li G., Shen G., Zhang P. and Xu Y. (2018) Methylation of bone SOST impairs SP7, RUNX2, and ER transactivation in patients with postmenopausal osteoporosis. Biochem. Cell Biol. Sep 26, Epub ahead of print 10.1139/bcb-2018-0170 30257098

[39] Avila T., Andrade A. and Felix R. (2006) Transforming growth factor-beta1 and bone morphogenetic protein-2 downregulate CaV3.1 channel expression in mouse C2C12 myoblasts. J. Cell. Physiol. 209, 448–456 10.1002/jcp.20743 16883604

[40] Kitoh H. and Ishiguro N. (2007) Molecular mechanism in the differentiation of chondrocytes. Clin. Calcium 17, 493–498 17404477

[41] Fischer D., Sun X., Williams A.B., Gang G., Pritts T.A., James J.H. (2001) Dantrolene reduces serum TNFalpha and corticosterone levels and muscle calcium, calpain gene expression, and protein breakdown in septic rats. Shock 15, 200–207 10.1097/00024382-200115030-00007 11236903

[42] Fang C.H., Sun X., Li B.G., Fischer D.R., Pritts T.A., Penner G. (2000) Burn injuries in rats upregulate the gene expression of the ubiquitin-conjugating enzyme E2(14k) in skeletal muscle. J. Burn. Care. Rehabil. 21, 528–534 10.1097/00004630-200021060-00010 11194807

[43] West A.B., Dawson V.L. and Dawson T.M. (2005) To die or grow: Parkinson’s disease and cancer. Trends Neurosci. 28, 348–352 10.1016/j.tins.2005.05.002 15913799

[44] Rhyu D., Park J., Sharma B.R. and Ha H. (2012) Role of reactive oxygen species in transforming growth factor-beta1-induced extracellular matrix accumulation in renal tubular epithelial cells. Transplant. Proc. 44, 625–628 10.1016/j.transproceed.2011.12.054 22483454

