# Supplementary figures and images for: TGF-β prevents the denervation-induced reduction of bone formation and promotes the bone regeneration through inhibiting ubiquitin-proteasome pathway
Source: Biosci Rep. 2019 May 14;39(5):BSR20190350. doi: 10.1042/BSR20190350 (PMC6522721; doi:10.1042/BSR20190350)

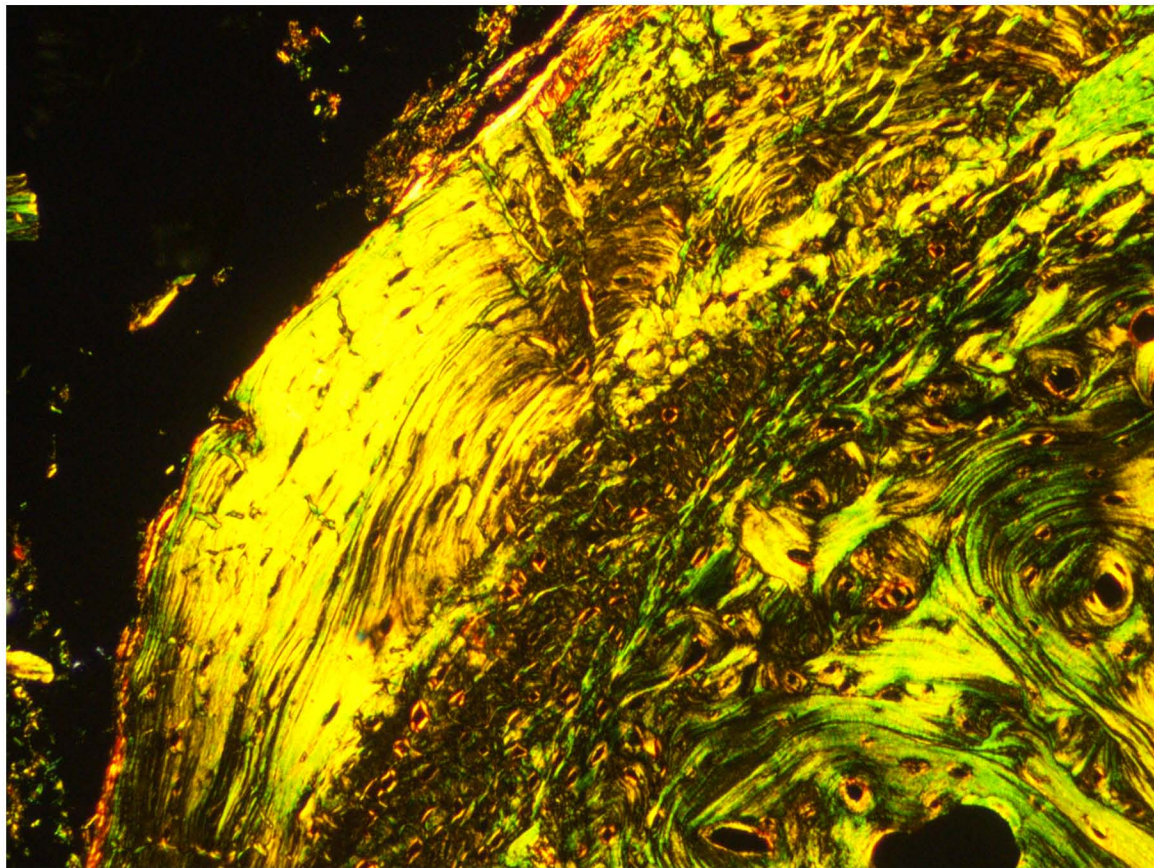

×200

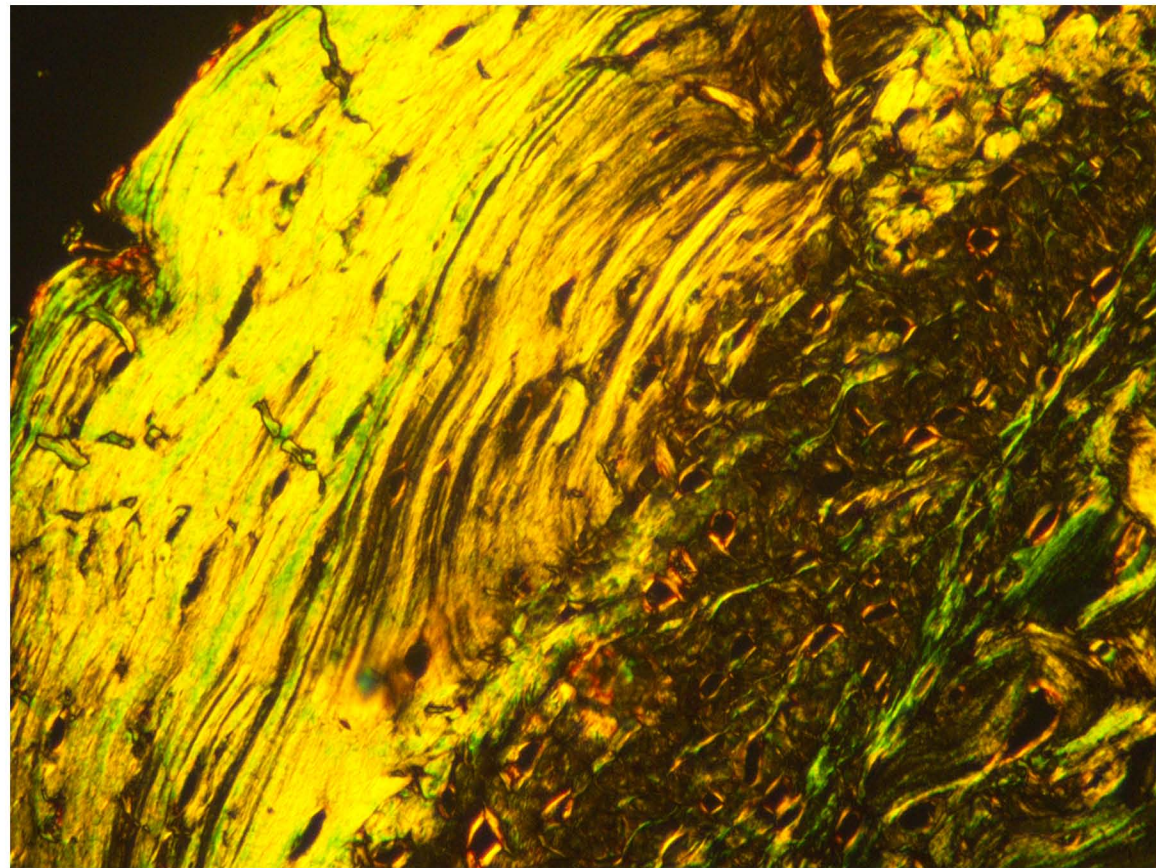

×400

Supplement: Supplementary file 1 [file BSR-2019-0350_suppS1.pdf]
